# A Retrospective Study about the Impact of Switching from Nested PCR to Multiplex Real-Time PCR on the Distribution of the Human Papillomavirus (HPV) Genotypes

**DOI:** 10.3390/medicina55080418

**Published:** 2019-07-30

**Authors:** Raffaele Del Prete, Luigi Ronga, Grazia Addati, Raffaella Magrone, Angela Abbasciano, Domenico Di Carlo, Luigi Santacroce

**Affiliations:** 1 Section of Microbiology, Interdisciplinary Department of Medicine (DIM), School of Medicine, University of Bari “Aldo Moro”, 70124 Bari, Italy; 2 UOC Microbiology and Virology, Azienda Ospedaliera-Universitaria Policlinico of Bari, 70124 Bari, Italy; 3 Pediatric Clinical Research Center “Romeo and Erica Invernizzi”, University of Milan, 20157 Milan, Italy; 4 Department of Biology and Biotechnology, University of Pavia, 27100 Pavia, Italy; 5 Ionian Department, University of Bari “Aldo Moro”, 70100 Bari, Italy

**Keywords:** HPV, sexually transmitted diseases (STDs), laboratory methods, PCR, genotypes, surveillance, epidemiology

## Abstract

Background and objectives: Human papillomavirus (HPV) is the most prevalent etiological agent of viral sexually-transmitted infection. This study retrospectively evaluated the impact of a switch to a real-time PCR assay in the HPV prevalence and genotypes distribution by a quasi-experimental before-and-after approach. Materials and Methods: In total, 1742 samples collected from 1433 patients were analyzed at the UOC Microbiology and Virology of Policlinico of Bari, Italy. HPV DNA detection was performed using initially nested PCR and subsequently multiplex real-time PCR assay. Results: Statistically significant difference in HPV overall prevalence after the introduction of the real-time assay was not detected (48.97% vs. 50.62%). According to different extraction-DNA amplification methods, differences were observed in the prevalence rates of HPV-45, 68, 40, 42, and 43. The lowest prevalence for HPV-45 was observed in the Magna Pure-Real Time PCR group, while HPV-68, 40, 42, and 43 were less observed in the Qiagen-Real Time PCR group. After, a multivariate logistic regression, an increase in the prevalence of HPV-42 (aOR: 4.08, 95% CI: 1.71–9.73) was associated with the multiplex real-time PCR assay. Conclusions: Although this study is a not a direct comparison between two diagnostic methods because it has a sequential structure, it serves to verify the impact of a new molecular assay on HPV distribution. Moreover, the stability of HPV prevalence over time suggests that the population composition and the behavioral variables did not likely change during the observation period. Our study proposes that the introduction of a molecular test for HPV detection may be related to changes of HPV genotypes distribution.

## 1. Introduction

Human papillomavirus (HPV) is still the most prevalent viral sexually-transmitted infection either in men or women. Clinically, it is characterized by a wide spectrum of manifestations, including premalignant lesions that regress spontaneously and malignant lesions evolving to cervical cancer (CC) [1].

Worldwide, although cervical screening programs have contributed to a decrease in the incidence, CC continues to be the second most common cancer among women, with an estimated 266,000 deaths for year [2].

Nevertheless, the use of combined tests to detect the presence of HPV DNA together with conventional cytology examination has been shown to greatly improve the ability to detect the pre-cancerous states [3,4]. Nowadays, with the aim of detecting HPV DNA a wide variety of laboratory diagnostic methods characterized by different grade of sensitivity and specificity are developed [5,6,7,8].

Until a few years ago, PCR followed by the nucleic acid hybridization techniques were used to detect HPV genotypes. When compared to conventional cytology, these techniques provided more detailed information regarding HPV genotypes [9].

Afterwards, for HPV diagnosis real-time PCR techniques were introduced. Their performance has significantly improved both the hands-on time and decreased contamination rates.

This study covers three years of routine diagnostic data on HPV DNA and aims to retrospectively evaluate the impact on HPV prevalence and HPV genotypes distribution of a switch from a nested-based PCR to a real-time based PCR on genital samples collected from patients in the Apulia region. To address this issue, a quasi-experimental approach for evaluating the effect of the real-time based PCR on the HPV prevalence and the prevalence of the single viral genotypes was based on the application of multiple logistic regression.

## 2. Materials and Methods

### 2.1. Clinical HPV Isolates and Patient Population Characteristics

From January 2012 to December 2014, 1742 consecutive samples, including 1605 cervico-vaginal swabs from 1328 females and 137 urethral swabs from 105 males were collected. Multi samples for some patients were due to retesting in different times.

Specimens were transferred to the laboratory of Molecular Biology, U.O.C. Microbiology and Virology, Azienda Ospedaliera-Universitaria, Policlinico of Bari, where they were analyzed.

All procedures performed in studies involving human participants were in accordance with the ethical standards.

Sample information (date of sampling, ward, type of specimen, testing results) together with the data of patients for whom molecular testing was performed (i.e., age and sex) were recorded in an anonymous database by changing sensitive data into alphanumeric codes. No clinical data associated with these specimens were available.

All procedures performed in studies involving human participants were in accordance with the ethical standards of the institutional and/or national research committee and with the 1964 Helsinki declaration and its later amendments or comparable ethical standards. For this type of study, formal consent is not required. This study was approved by Ethics Committee (No. 5481, 13 December 2017) Azienda Ospedaliero-Universitaria “Consorziale Policlinico,” Bari.

### 2.2. Treatment of Samples

A total of 2 mL of phosphate-buffered saline (pH 7.4) (Sigma-Aldrich, Milano, Italy) was added to cervical-vaginal swabs, collected by a rigid cotton-tipped swab applicator (Nuova Aptaca, Cannelli, Italy), and vortexed. Then, 1 mL of phosphate buffered saline (Sigma, Milano, Italy) was added to urethral swabs and vortexed. Finally, all samples were transferred to microcentrifuge tubes and they were stored at −20 °C until processing. To extract viral nucleic acids, microcentrifuge tubes were centrifuged at rcf = 15,700× *g* for 15 min at 7 °C. The majority of supernatant was discarded but 200 µL of supernatant was retained to resuspend the pellet.

### 2.3. DNA Isolation (QIAcube System vs. MagNa Pure 96 System)

From January 2012 to December 2013, DNA extraction was performed by automated QIAcube System (Qiagen, Hilden, Germany), following the manufacturer’s protocols.

From January 2014 to December 2014, the vilrral nucleic acids were extracted from the resuspended pellet using the automated MagNa Pure 96 system (Roche Diagnostics GmbH, Mennheim, Germany) according to the manufacturer’s instructions.

### 2.4. DNA Amplification (Nested-PCR vs. Multiplex Real-Time PCR)

From January 2012 to June 2013, the extracted DNA samples were subject to a nested polymerase chain reaction (PCR) amplification, using Ampliquality HPV-HS Bio Kit (AB Analitica, Padova, Italy) following the manufacturer’s instructions.

The method provides for a first amplification of the viral genome L1 region, followed by a nested PCR with biotinylated primers. PCR products were analyzed using 3% agarose gel electrophoresis with ethidium bromide to display DNA under ultraviolet light. Subsequently, PCR products were typed by using Reverse Line Blot Hybridization Ampliquality HPV-Type Kit (AB Analitica, Padova, Italy).

To assess the suitability of extracted DNA, the thiosulfate sulfurtransferase (TST) gene region (202 bp) was amplified at the first amplification.

From July 2013 to December 2014, the extracted DNA samples were subject to multiplex real-time PCR (mRT-PCR) by Anyplex^TM^ II HPV 28 Detection System (Seegene, Seoul, Korea), which targets the viral L1 region and provides simultaneous detection and genotyping of 28 HPV-types. Briefly, the detection consists of two PCR reactions (panel A and B). The panel A includes 14 high-risk HPV (HR/HPV)-types, while the panel B includes 5 HR and 9 low-risk (LR)-types. PCR was performed on the CFX96 Real-Time PCR system (Bio-Rad, Hercules, CA, USA).

### 2.5. Statistical Analysis

Differences in HPV prevalence were evaluated by Chi-Squared test and Fisher’s test as appropriate. *p*-values were corrected by Benjamini and Hochberg’s (BH) procedure with False Discovery Rate (FDR) <1% [10]. Pairwise comparison was performed on each statistically significant combination group of extraction and amplification methods by Fisher’s test and BH’s correction with FDR <1%.

To assess the association of the real-time assay on the prevalence of the HPV overall infection and the prevalence of each HPV genotype (dependent variables) on the analyzed samples (samples dataset), logistic regression analysis was performed. Due to the lack of birth date for 102 patients, missing ages were imputed by multiple imputation by fully conditional specification implemented in the Multivariate Imputation by Chained Equations (MICE) package implemented in the environment R. A predictive mean matching imputation model was specified on the assumption of missing at random ages and the number of iterations was set to 20. In particular, 50 imputed data sets were generated. For each dataset, a logistic regression model was generated, and the 50 models were pooled together by the function pool of the package mice. Globally, 27 logistic regression models were evaluated. All *p*-values collected from the logistic regression models were corrected for multiple comparisons by BH procedure with FDR < 1%.

Logistic regression analysis is based on the assumption of independence of the variables. To verify this assumption, a reduced dataset only containing the first sample for each patient (patient dataset) was generated and all analyses were repeated on it. Odds ratio estimations of the logistic regression models based on the samples and the patients’ datasets were compared.

Calculations of all statistical tests were performed by the open source environment R [11].

## 3. Results

From 1 January 2012 to 31 December 2014, 1742 samples (1605 cervical-vaginal and 137 urethral swabs) from 1433 patients (1328 female and 105 male patients, Female to Male ratio = 12.64) were analyzed.

During the observation period, the number of analyzed samples increased with time from a minimum value of 306 in 2012 to an intermediate value of 666 and to a maximum value of 770 in 2014. Moreover, after the introduction of the real-time assay an increase of analyzed cervico-vaginal swabs (87.66% vs. 94.12%) and a percentage decrease of urethral swabs (12.34% vs. 5.88%) was observed.

From January 2012 to the end of June 2013, 535 samples were extracted by QIAcube System (Qiagen, Hilden, Germany) followed by a nested-PCR technique. From July 2013 to the end of December 2013, 437 specimens were extracted by QIAcube followed by mRT-PCR. From January to December 2014, 770 samples were extracted by MagNa Pure 96 system (Roche) and amplified by mRT-PCR. The HPV prevalence in the three groups was 48.97% (262), 49.65% (217), and 51.16% (394), respectively (Chi-Squared test *p*-value = 0.719).

After stratification for the three extraction-amplification combinations, significant differences in HPV types prevalence rates were observed for HPV-45, 68, 40, 42, and 43. In particular, the lowest prevalence for HPV-45 was observed in the Magna Pure-Real Time PCR group (0.00%) while HPV-68, 40, 42, and 43 were less observed in the Qiagen-Real Time PCR group (0.37%, 0.00%, 2.06%, and 0.00%, respectively) (Table 1).

In particular, after pairwise comparison, QIAcube-real time combination resulted in higher HPV-45 prevalence compared to Magna Pure-Real Time PCR (2.99% vs. 0.00%), in lower HPV-68, HPV-40, HPV-42 prevalence rates compared to both Magna Pure-Real Time PCR and QIAcube-Nested PCR (HPV-68: 0.37% vs. 3.20% and 0.37% vs. 3.90%, respectively; HPV-40: 0.00% vs. 1.83% and 0.00% vs. 2.08%, respectively; HPV-42: 2.06% vs. 10.53% and 2.06% vs. 13.25%, respectively) and in lower HPV-40 prevalence compared to QIAcube-Nested PCR (0.00% vs. 1.82%).

On logistic regression analysis, the introduction of real-time assay was associated with an increase of HPV-42 (aOR: 4.08, 95% CI: 1.71–9.73) (Table 2). Moreover, the logistic regression analysis did not reveal the presence of background trends. The comparison of the estimates of the odds ratios of the models built on the samples dataset and the patients dataset revealed absolute differences <1 in the majority of cases (Table 2).

## 4. Discussion

This study evaluated the HPV positivity rates and the HPV genotypes distribution before and after the switch from a nested-based PCR to a real-time PCR on a three-year observation window. Multivariate analysis showed that the HPV prevalence was not affected by the introduction of the new real-time assay. The sensitivity of a nested PCR compared to a real-time amplification method was analyzed by several authors, but the conclusions were not unique [12,13,14].

On the other hand, some differences in the distribution of HPV genotypes were detected. In particular, the pairwise comparison showed some statistically significant differences in the distribution of HPV-45, 68, 40, 42, and 43. The analysis also highlighted that these differences were due to the QIAcube-Real Time PCR group. Logistic regression analysis only confirmed that the Real Time introduction was associated with an increase of the prevalence of HPV-42 (aOR: 4.08, 95%CI: 1.71–9.73).

Currently, more than 100 distinct molecular tests are available on the global market for the detection of HPV DNA [15]. However, despite the approving of several tests for clinical use in USA and Europe, some tests detect a pool of 12 HPV genotypes (HPV 16, 18, 31, 33, 35, 39, 45, 51, 52, 56, 58, and 59) while other tests also include HPV 66 and 68 [15]. On the other hand, some commercially available tests allow specific HPV genotyping [16]. Some studies reported that genotyping techniques might be useful to improve the triage and follow up of HPV infected women [17]. In fact, some authors suggested that the genotypes 16/18 are related to a more elevated progressive risk [18,19].

An important source of variations is that the detection of specific HPV genotypes may be affected by the different sensitivities of the currently available genotyping methods.

In a study of Del Pino et al. [16] several genotyping tests (Anyplex^TM^ II HPV 28 Detection System, Linear Array HPV genotyping test, Gp5+/6+ PCR-EIA-RH, CLART HPV2 Assay) were compared with Hybrid Capture 2 and a concordance about 80% or higher was reported. In particular, the comparison of the genotype distribution between Anyplex^TM^ and Linear Array, Gp5+/6+ and CLART2, respectively, showed completely different genotypes in five (4.0%), two (2.3%), and three (2.9%) samples.

In a study of Lim et al. [20], Anyplex^TM^ was compared with MolecuTech REBA HPV-ID and HPV DNAChip and the percentage of HPV genotype agreement ranged from 93.7% to 100.0%. However, relatively high rates of discordance between the three assays were reported for HPV 31, 42, and 44. The authors also examined the performance of the assays in the detection of five common HPV genotypes (HPV 16, 18, 45, 52, and 58) and reported that the sensitivity rates varied according to the detection method. In particular, MolecuTech showed a low sensitivity for HPV 52 (42.9%) while Anyplex^TM^ and HPV DNAChip had low sensitivities for HPV 45 (25.0%).

Comparison of Anyplex^TM^ with Euroarray on 150 samples by Latsuzbaia et al. [21] showed a Kappa concordance below 0.7 for HPV 40, 68, 73, and above 0.95 for HPV 11, 16, 18, 33, and 59. Interestingly, a statistically significant difference between assays was detected for HPV 42 genotype since it was more frequently detected by Anyplex^TM^ assay.

Marcuccilli et al. [22] reported that Anyplex^TM^, compared with HPV Sign Genotyping Test, detected more high risk and low risk genotypes. In particular, better agreements were reported for HPV 16, 18, 35, and 70. Statistically significant differences were reported for HPV 31, 51, 52, 53, 56, 58, 59, 66, 73, 6, 42, 44, 54, and 61. These genotypes, except HPV 73, were more frequently detected by Anyplex^TM^ assay.

Differences in HPV genotype detection were also reported by Estrade et al. [23] after comparison of Anyplex^TM^ with PGMY-CHUV assay. In particular, HPV 40, 42, 54, and 68 were significantly more frequently detected by AnyplexTM. On the contrary, HPV 51 was more frequently detected by the PGMY-CHUV assay.

The sequential design does not permit to infer the different sensitivities or specificities of the two diagnostic tests. However, the quasi-experimental approach of the study may be advantageous to acquire some preliminary information to better design subsequent studies or to speculate regarding the clinical usefulness of a new diagnostic assay in a relatively cheap manner. In particular, the latter option may be of some interest in a lack of funds context. To our knowledge, this is the first time that a quasi-experimental before-and-after approach has been applied to the evaluation of a new assay for the HPV evaluation. In particular, the data suggest that the introduction of Real-Time PCR did not affect the HPV prevalence, but it was associated with modification of the distribution of HPV-42. Other studies will be needed to clarify the reasons for these variations. However, other limits of this study must be considered. In particular, the temporal window is quite short and the influence of pluriannual trends cannot be excluded. Moreover, the composition of the population analyzed is not known and the differences in the distribution of the HPV genotypes may reflect differences in the behaviors or risk factors of the analyzed patients. However, the presence of background trends was also excluded by the logistic regression analysis and this makes it possible to assume that the behavioral and social parameters of the analyzed population have not changed over time.

Despite these limits, such study suggests to carefully evaluate the introduction of a new type test for HPV also regarding the impact on the distribution of HPV genotypes. In particular, it may be considered as a potential confounding factor for evaluation of public health programs to control HPV spreading in the populations. At the same time, our study highlights the importance to carefully evaluate temporal dynamics with multivariate methods. Related to this, the accumulation of more rich surveillance data should be encouraged to ensure that demographic shifts in infection patterns could be accurately monitored.

## 5. Conclusions

Having in mind that any technical innovation is usually related to new perspectives for both clinical and technical development over time, the results obtained suggest that the temporal distribution of HPV genotypes might be influenced by the diagnostic methods that are performed.

This study is not a direct comparison of two diagnostic methods because it has a sequential design. In summary, with univariate analysis significant differences were observed in the prevalence rates of HPV-45, 68, 40, 42, and 43. The lowest prevalence for HPV-45 was observed in the Magna Pure-Real Time PCR group, while HPV-68, 40, 42, and 43 were less observed in the Qiagen-Real Time PCR group.

With logistic regression analysis, the introduction of the real-time assay was associated with an increase in HPV-42. Furthermore, the logistic regression analysis did not reveal the presence of underlying trends. It is possible that the difference in the HPV-42 distribution detected after the switch to the real-time test could be related to the different sensitivities of the two diagnostic assays or to the presence of cross-reactions.

An important limitation of this kind of study is the potential confounding effect of some variables (secular trends, changes in population composition, or behavioral risks). Unfortunately, these data were not available for the analysis. However, despite the increase in number of subjects tested, the stability of HPV prevalence over time suggests that the population composition and the behavioral variables did not likely change during the observation period.

Therefore, this parameter should be taken into the consideration when a multivariate statistical model is represented, and further clinical studies could increase knowledge on the prevalence of HPV based on the use of different methods.

## Figures and Tables

**Table 1:** Human papillomavirus (HPV) genotype prevalence in the three different combination groups of extraction and amplification methods.

HPV Genotype	QIAcube-Nested PCR *n*, (%)	QIAcube-Real Time PCR *n*, (%)	Magna Pure-Real Time PCR *n*, (%)	*p*-Value	BH-Correction
HPV-16	64 (8.31%)	55 (10.28%)	47 (10.76%)	0.29	NS
HPV-18	21 (2.73%)	10 (1.87%)	9 (2.06%)	0.58	NS
HPV-31	68 (8.83%)	36 (6.73%)	25 (5.72%)	0.11	NS
HPV-33	16 (2.08%)	10 (1.87%)	7 (1.60%)	0.85	NS
HPV-35	10 (1.30%)	7 (1.31%)	6 (1.37%)	1.00	NS
HPV-39	20 (2.60%)	6 (1.12%)	11 (2.52%)	0.14	NS
HPV-45	10 (1.30%)	16 (2.99%)	0 (0.00%)	<0.01	S
HPV-51	30 (3.90%)	7 (1.31%)	17 (3.89%)	0.01	NS
HPV-52	19 (2.47%)	10 (1.87%)	10 (2.29%)	0.80	NS
HPV-53	70 (9.09%)	26 (4.86%)	34 (7.78%)	0.01	NS
HPV-56	31 (4.03%)	14 (2.62%)	13 (2.97%)	0.36	NS
HPV-58	30 (3.90%)	24 (4.49%)	15 (3.43%)	0.72	NS
HPV-59	21 (2.73%)	8 (1.50%)	11 (2.52%)	0.33	NS
HPV-66	30 (5.32%)	2 (3.18%)	14 (4.81%)	0.17	NS
HPV-68	30 (3.90%)	2 (0.37%)	14 (3.20%)	<0.01	S
HPV-73	22 (82.86%)	20 (3.74%)	15 (3.43%)	0.66	NS
HPV-82	3 (0.39%)	3 (0.56%)	3 (0.69%)	0.76	NS
HPV-6	46 85.97%)	25 (4.67%)	16 (3.66%)	0.20	NS
HPV-11	18 (2.34%)	7 (1.31%)	2 (0.46%)	0.03	NS
HPV-40	16 (2.08%)	0 (0.00%)	8 (1.83%)	<0.01	S
HPV-42	102 (13.25%)	11 (2.06%)	46 (10.53%)	<0.01	S
HPV-43	14 (1.82%)	0 (0.00%)	4 (0.92%)	<0.01	S
HPV-44	19 (2.47%)	3 (0.56%)	10 (2.29%)	0.02	NS
HPV-54	52 6.75	29 (5.42%)	25 (5.72%)	0.60	NS
HPV-61	27 3.51	12 (2.24%)	23 (5.26%)	0.04	NS
HPV-70	12 1.56	11 (2.06%)	6 (1.37%)	0.70	NS

NS: Non-Significant; S: Significant. *p*-values were calculated on a 2 × 3 matrix by Fisher’s test and then corrected by Benjamini and Hochberg’s correction (False Discovery Rate (FDR) <1%).

**Table 2:** Evaluation by logistic regression models of introduction of real time assay on HPV and HPV genotypes detection.

Dependent Variable	Samples Dataset Odds Ratio (95% Confidence Interval)					Patients Datase tOdds Ratio (95% Confidence Interval)				
	Age	Sex (M vs. F)	Year	DNA Extraction	Test Type	Age	Sex (M vs. F)	Year	DNA Extraction	Test Type
*HPV-16 →*	0.99 (0.97–1.01)	0.69 (0.34–1.4)	1.35 (0.75–2.44)	1.81 (0.88–3.71)	0.84 (0.51–1.39)	0.98 (0.96–1)	0.86 (0.4–1.84)	1.11 (0.56–2.18)	1.44 (0.64–3.23)	1.05 (0.58–1.89)
*HPV-18 →*	0.98 (0.94–1.01)	0.7 (0.16–2.97)	0.7 (0.19–2.55)	0.55 (0.12–2.5)	1.12 (0.34–3.67)	0.97 (0.94–1.01)	0.85 (0.2–3.65)	0.93 (0.24–3.56)	0.74 (0.15–3.59)	1.02 (0.31–3.39)
*HPV-31 →*	**0.95 (0.93–0.97)**	0.7 (0.32–1.57)	1.65 (0.8–3.41)	1.1 (0.46–2.63)	0.55 (0.3–1)	**0.96 (0.94–0.98)**	0.74 (0.29–1.89)	1.56 (0.69–3.53)	0.87 (0.33–2.33)	0.54 (0.27–1.1)
*HPV-33 →*	0.97 (0.93–1.01)	0.39 (0.05–2.91)	2.14 (0.52–8.76)	1.77 (0.33–9.45)	0.56 (0.18–1.69)	0.97 (0.93–1.01)	0.5 (0.07–3.77)	2 (0.46–8.61)	1.96 (0.34–11.26)	0.61 (0.19–1.97)
*HPV-35 →*	0.97 (0.92–1.01)	0 (0–Inf)	2.68 (0.51–14.14)	3.27 (0.46–23.19)	0.56 (0.17–1.86)	0.97 (0.92–1.02)	0 (0–Inf)	1.83 (0.29–11.32)	1.65 (0.18–14.89)	0.57 (0.12–2.57)
*HPV-39 →*	**0.92 (0.89–0.96)**	0.37 (0.05–2.74)	0.22 (0.02–1.96)	0.23 (0.02–2.39)	5.24 (0.67–41.05)	**0.89 (0.84–0.94)**	0.45 (0.06–3.46)	0.2 (0.02–1.88)	0.25 (0.02–2.64)	4.5 (0.57–35.81)
*HPV-45 →*	1.01 (0.97–1.05)	1.71 (0.48–6.04)	1.74 (0.6–5.04)	0 (0–Inf)	0 (0–Inf)	1.01 (0.96–1.06)	2 (0.42–9.44)	1.06 (0.27–4.1)	0 (0–Inf)	0 (0–Inf)
*HPV-51 →*	**0.94 (0.91–0.98)**	0.25 (0.03–1.82)	0.95 (0.19–4.82)	0.99 (0.18–5.57)	2.73 (0.79–9.48)	0.95 (0.92–0.99)	0.33 (0.04–2.48)	1.1 (0.22–5.57)	0.92 (0.16–5.32)	2.09 (0.59–7.39)
*HPV-52 →*	1.01 (0.97–1.04)	0 (0–Inf)	0.82 (0.23–2.97)	0.92 (0.2–4.18)	1.26 (0.39–4.08)	1.01 (0.98–1.04)	0 (0–Inf)	0.98 (0.27–3.59)	0.92 (0.2–4.32)	0.99 (0.3–3.26)
*HPV-53 →*	0.98 (0.96–1)	0.82 (0.37–1.82)	0.6 (0.25–1.47)	0.53 (0.2–1.43)	2.22 (1.01–4.9)	0.98 (0.96–1)	0.83 (0.33–2.13)	0.69 (0.27–1.82)	0.61 (0.21–1.78)	1.89 (0.81–4.43)
*HPV-56 →*	0.99 (0.97–1.02)	0.84 (0.26–2.77)	0.64 (0.18–2.22)	0.49 (0.12–2)	1.67 (0.54–5.21)	1 (0.97–1.03)	1.13 (0.34–3.76)	0.64 (0.16–2.64)	0.49 (0.1–2.36)	2.06 (0.58–7.34)
*HPV-58 →*	1 (0.97–1.02)	0.17 (0.02–1.27)	1.91 (0.8–4.53)	1.68 (0.58–4.91)	0.52 (0.24–1.09)	1 (0.97–1.02)	0.24 (0.03–1.78)	1.74 (0.66–4.57)	1.52 (0.46–4.98)	0.56 (0.24–1.31)
*HPV-59 →*	0.98 (0.94–1.01)	0.78 (0.18–3.31)	0.42 (0.08–2.19)	0.36 (0.06–2.25)	2.51 (0.54–11.61)	0.99 (0.95–1.02)	0.51 (0.07–3.81)	0.25 (0.03–2.18)	0.24 (0.02–2.41)	4.58 (0.58–36.14)
*HPV-66 →*	0.97 (0.94–0.99)	0.54 (0.17–1.76)	1.04 (0.36–3.04)	0.91 (0.27–3.04)	1.41 (0.59–3.4)	0.97 (0.95–1)	0.47 (0.11–1.97)	1.03 (0.34–3.16)	1.01 (0.28–3.57)	1.43 (0.56–3.66)
*HPV-68 →*	1 (0.97–1.03)	0 (0–Inf)	0 (0–Inf)	0 (0–Inf)	26,437,379.16 (0–Inf)	0.98 (0.95–1.02)	0 (0–Inf)	0 (0–Inf)	0 (0–Inf)	27,473,819.35 (0–Inf)
*HPV-73 →*	0.97 (0.94–1)	0.9 (0.32–2.58)	1.16 (0.46–2.94)	1.35 (0.42–4.28)	0.68 (0.3–1.57)	0.98 (0.95–1.01)	1.08 (0.37–3.12)	1.33 (0.52–3.39)	1.63 (0.49–5.41)	0.58 (0.25–1.37)
*HPV-82 →*	0.95 (0.87–1.03)	1.6 (0.19–13.52)	0.93 (0.06–15.13)	1.59 (0.06–40)	1.5 (0.15–14.6)	0.95 (0.88–1.03)	1.83 (0.21–15.6)	1.03 (0.06–16.92)	1.54 (0.06–38.94)	1.44 (0.15–14.1)
*HPV-6 →*	0.98 (0.96–1.01)	2.39 (1.24–4.61)	1.36 (0.58–3.19)	0.78 (0.27–2.19)	0.63 (0.3–1.34)	0.99 (0.96–1.01)	2.23 (1.01–4.93)	1.19 (0.43–3.27)	0.81 (0.25–2.65)	0.98 (0.41–2.34)
*HPV-11 →*	1 (0.96–1.04)	**10.21 (4.2–24.82)**	1.83 (0.32–10.34)	0.26 (0.03–2.61)	0.29 (0.05–1.61)	1 (0.94–1.05)	**11.4 (3.41–38.09)**	1.01 (0.14–7.47)	0.12 (0.01–2.22)	0.3 (0.03–3.37)
*HPV-40 →*	0.99 (0.94–1.03)	1.5 (0.34–6.6)	0.93 (0–Inf)	0.83 (0–Inf)	16,082,537.86 (0–Inf)	0.98 (0.93–1.03)	1.04 (0.13–8.11)	0.91 (0–Inf)	1.1 (0–Inf)	18,565,563.58 (0–Inf)
*HPV-42 →*	**0.97 (0.96–0.99)**	0.36 (0.13–1.01)	1.28 (0.38–4.29)	1.08 (0.3–3.81)	**4.08 (1.71–9.73)**	0.97 (0.95–0.99)	0.38 (0.12–1.23)	1.2 (0.34–4.25)	1.15 (0.3–4.31)	**4.31 (1.67–11.1)**
*HPV-43 →*	0.98 (0.94–1.03)	2.23 (0.49–10.05)	0.9 (0–Inf)	0.41 (0–Inf)	21,853,475.78 (0–Inf)	0.99 (0.94–1.04)	2.87 (0.62–13.23)	0.87 (0–Inf)	0.34 (0–Inf)	9,855,453.89 (0–Inf)
*HPV-44 →*	1.02 (0.99–1.06)	1.03 (0.24–4.45)	0.61 (0.05–6.85)	0.54 (0.04–6.84)	5.44 (0.69–42.88)	1.02 (0.98–1.06)	1.73 (0.39–7.69)	0.7 (0.06–7.9)	0.5 (0.04–6.72)	3.45 (0.42–28.42)
*HPV-54 →*	0.98 (0.96–1)	0.61 (0.24–1.55)	1.76 (0.8–3.86)	1.48 (0.59–3.76)	0.72 (0.38–1.36)	0.98 (0.95–1)	0.4 (0.1–1.67)	1.08 (0.4–2.89)	1.18 (0.38–3.68)	1.17 (0.51–2.71)
*HPV-61 →*	1.02 (0.99–1.04)	0.44 (0.1–1.82)	1.51 (0.45–5.06)	2.37 (0.62–9.04)	2.05 (0.82–5.12)	1 (0.97–1.03)	0.32 (0.04–2.39)	2.34 (0.55–10.06)	3.11 (0.63–15.39)	1.54 (0.56–4.26)
*HPV-70 →*	1.04 (1–1.07)	1.52 (0.44–5.2)	2.23 (0.64–7.83)	1.82 (0.37–9.04)	0.46 (0.15–1.4)	1.05 (1.01–1.09)	2.25 (0.49–10.4)	1.03 (0.17–6.41)	0.64 (0.07–5.73)	1.01 (0.18–5.6)
*HPV →*	**0.98 (0.97–0.99)**	0.83 (0.57–1.22)	1.24 (0.86–1.79)	1.17 (0.75–1.81)	0.84 (0.6–1.16)	0.98 (0.97–0.99)	0.84 (0.54–1.31)	**1.11 (0.74–1.66)**	1.08 (0.67–1.75)	0.92 (0.64–1.33)

Bolded: Statistically significant after BH’s correction. Each variable in the first column was the dependent variable (Absence vs. Presence) of the model while the independent variables were the Age (linear variable), the Sex (Male vs. Female), the Year of testing (linear variable), the DNA Extraction method (Qiagen vs. Magna Pure) and the Test Type (Real-Time PCR assay vs. Nested assay). Each regression analysis was performed on both the samples and the patient datasets.
